# Antioxidant effect of leaf extracts from *Cressa cretica* against oxidation process in soybean oil

**DOI:** 10.1002/fsn3.396

**Published:** 2016-06-18

**Authors:** Afsaneh Afshari, S. Zahra Sayyed‐Alangi

**Affiliations:** ^1^ Department of Food Science and TechnologyAzadshahr BranchIslamic Azad UniversityAzadshahrIran; ^2^ Department of ChemistryAzadshahr BranchIslamic Azad UniversityAzadshahrIran

**Keywords:** Antioxidant activity, *Cressa cretica*, oxidation, soybean oil

## Abstract

Phenolic compounds from *Cressa cretica* leaves were extracted using different solvents (water and ethanol 70%) during 3–24 h by immersion method and were determined according to the Folin–Ciocalteu method. The antioxidant activities of the extracts were investigated by 1,1‐diphenyl‐2‐picryl hydrazyl (DPPH) radical scavenging, ferric reduction activity potential and total antioxidant capacity assays compared with synthetic antioxidant namely BHT. The results were shown that the most total phenol content was outcome of the ethanolic extract after 18 h (68.512 ± 0.36^ ^mg gallic acid/g dry extract) extraction. The results of different assays for determination of antioxidant potential of the extracts as well as their EC_50_ values were indicated antioxidant activities in order: BHT>ethanolic extract>aqueous extract. Also, the antioxidant activities were enhanced with increasing of the extracts and BHT concentrations. The results of Peroxide value (PV) and Thiobarbituric acid (TBA) tests were revealed that the ethanolic extract with various concentrations (200–1000 ppm) were suppressed the oxidation of soybean oil. The highest oxidation inhibitor was belonged to 1000 ppm concentration of the ethanolic extract so that can be good alternative for BHT.

## Introduction

*Cressa cretica* Linn belongs to the family *Convolvulaceae* which are weeds of pistachio orchards. This plant is distributed throughout middle east specially Iran, India, Timor, and Australia (Satakopan and Karandikar 1961; Khan and Aziz 1998; Shahat et al. 2004; Priyashree et al. 2010). *Cressa cretica* is a small branched shrub growing extensively in sandy and saline soils. That is clumpy and low‐growing but usually has erect stems covered white‐haired, green leaves (Khan and Aziz 1998; Shahat et al. 2004; Santiago‐Adame et al. 2015). Different parts of the plant have been claimed to be valuable in a wide spectrum of diseases. In earlier studies, *C. cretica* Linn flowers exhibit cytotoxic and anti‐inflammatory activity in vitro (Sunita et al. 2009). Also, *C. cretica* is reported to be antibilous, antituberculosis, and expectorant properties (Rizk and El‐Ghazaly 1995). The plant also possesses antitussive activity in experimental rats (Fukumoto and Mazza 2000; Scalbert et al. 2005; Hernández et al. 2009). Furthermore, the plant is applied as stomachic, tonic, and aphrodisiac purposes, enriches the, and is useful in constipation, leprosy, asthma, and urinary discharges and anthelmintic, in the treatment of diabetes, Alzheimers disease and general debility (Memon et al. 2007; Verma et al. 2013). It is also reported the fruits of *C. cretica* is a potential source of edible oil (Sabikhi and Sathish Kumar 2012). In addition of several health benefits which previously reported, *Cressa cretica* is an excellent source of phenolic compounds. Shahat et al. were found five flavonoids (quercetin, quercetin‐3‐*O*‐glucoside, kaempferol‐3‐*O*‐rhamnoglucoside, and rutin) from the aerial parts of *C. cretica* (Shahat et al. 2004).

Polyphenols are natural antioxidants possess characteristic properties, such as free‐radical scavenging and inhibition of oxidizing processes in the body (Hernández et al. 2009). Plant‐derived antioxidants are molecules which donate electrons or hydrogen atoms. These compounds are able to form less reactive antioxidant‐derived radicals, which are efficiently quenched by other electron or hydrogen sources to prevent cellular damage. Therefore, they help to delay and inhibit lipid oxidation, protect human cells against oxidative damage, leading to a reduced risk of several oxidative‐stress associated degenerative diseases, such as cancer, cardiovascular, or neurodegenerative diseases (Scalbert et al. 2005) and when added to foods tend to minimize rancidity, retard the formation of toxic oxidation products, help to maintain the nutritional quality and increase their shelf life (Fukumoto and Mazza 2000). Natural antioxidants, which are present in variable amounts in vegetables such as fruits, leaves, flowers, roots, grains, and seeds, have gained prominence as alternatives to synthetic antioxidants. Their ability to do this is based mainly on their phenol‐derived structure. Many attempts have been made to replace synthetic antioxidants by natural products in the stabilization of fats against oxidative rancidity. The emphasis given to natural antioxidants results from concerns over the toxicity of some synthetic antioxidants, such as BHA, BHT, and EQ and from the search findings which point to a relationship between bioactive dietary ingredients, like natural antioxidants, and their protection of cells from oxidative damage in the human body (Ahn et al. 2012).

Pryianka et al. (2015) were evaluated in vitro antioxidant activity of petroleum ether extract of *Cressa Cretica* by hydrogen peroxide radicals scavenging and 1,1‐diphenyl‐2‐picryl hydrazyl (DPPH) assay. Also, the antioxidant activities, the total phenolic content (TPC) and total flavonoid content (TFC) of methanolic and ethylacetate fractions of *Cressa Cretica* were investigated by Sunita et al. (2011). According to our knowledge, there are no studies about evaluation of the antioxidant activities of water and ethanol extracts of *Cressa cretica* leaves as well as its oxidative stability on oils. Therefore, the objective of this study was to evaluate of the effects of the water and ethanol solvents, and extraction time on the total phenolic content and antioxidant activity of *Cressa cretica* different extracts and its application in inhibition of oxidizing processes of soybean oil.

## Materials and Methods

### Materials

*Cressa cretica* was collected from Rafsanjan in Kerman province (south part of Iran) on September 2014. Pure soybean oil was purchased from Alia Golestan Company (Kordkooy, Iran). All other chemicals used in this study were analytical grade and purchased from chemical suppliers.

### Preparation of *Cressa cretica* aqueous and ethanolic extracts

After drying and produce *Cressa cretica* leaves powder, Phenolic extracts were obtained by immersion extraction using ethanol (70%) and water solvents with 1:10 ratio (dried plant: solvent w/v) from 3 to 24 h at ambient temperature. Each extract was filtered with Whatman No. 1 filter paper. Then, they were evaporated to dryness at 40°C in a rotary evaporator (RV10, IKA, Germany) (Arabshahi‐Delouee and Urooj 2007). Samples were placed in eppendrof tubes (2 mL), closed and kept under refrigerated conditions (4°C).

### Determination of TPC of the extracts

The total phenol content (TPC) was determined by the method of Slinkard and Singleton (Slinkard and Singleton 1977) involving Folin‐Ciocalteu reagent and gallic acid as standard. Extract solution containing 20 *μ*L extract was taken in a volumetric flask, 2 mL distilled water, and 100 *μ*L Folin‐Ciocalteu reagent were added and flask was shaken thoroughly. After 5 min, 300 *μ*L of solution 20% Na_2_CO_3_ was added. Then, the mixture was allowed to stand in a water bath at 40°C for 30 min with intermittent shaking. Absorbance was measured with a UV‐spectrophotometer (PG‐instrument‐Ltd, USA) at 760 nm. The same procedure was repeated to all standard gallic acid solutions and standard curve was obtained. The content of phenols in the test samples were found out from the standard curve and the results were expressed as mg gallic acid equivalents/g sample.

### DPPH radical scavenging method

The antioxidant activity of the extracts was measured by DPPH (2, 2‐Diphenyl‐1‐picrylhydrazyl) radical scavenging assay according to the method followed by Shimada et al. (Shimada et al. 1992). Different concentrations of each extracts (25–200 ppm) were added, at an equal volume, to methanolic solution of DPPH (1 mmol/L). The mixtures were well shaken and then placed in a dark room. After 30 min at room temperature, the absorbance was recorded at 517 nm. In the control sample, the extracts were replaced with 3 mL methanol. The experiment was repeated for three times. BHT was used as standard controls. The percentage inhibition of the DPPH radical was calculated according to the formula of Yen and Duh (1994).

I = [(AB‐AS)/AB] × 100.

where, I = DPPH inhibition (%), AB = absorbance of control sample (0 min), and AS = absorbance of a tested sample at the end of the reaction (after 30 min). EC_50_ values denote the concentration of sample, which is required to scavenge 50% of DPPH‐free radicals, measured at 517 nm (Aksoy et al. 2013).

### Ferric reducing antioxidant power assay

The ability of extracts to reduce iron (III) was evaluated using the method of Yildirim et al. (2001) (Yildirim et al. 2001). Samples (1 mL) were mixed with 2.5 mL of phosphate buffer (0.2 mol/L, pH 6.6) and 2.5 mL of potassium ferricyanide (K_3_Fe(CN)_6_; 10 g/L) and incubated for 30 min at 50°C. Then, 2.5 mL of trichloroacetic acid (10% w/v) was added to the solution and centrifuged for 10 min. Finally, 2.5 mL of supernatant was combined with 2.5 mL of distilled water and 0.5 mL FeCl_3_ (1 g/L). The absorbance of samples was measured at 700 nm. Higher absorbance means higher reducing power (Aksoy et al. 2013).

### Total antioxidant capacity

The assay was done using the method of Prieto et al. (1999) (Prieto et al. 1999). 0.1 mL of samples was mixed with 1 mL of reagent (0.6 mol/L sulfuric acid, 28 m mol/L sodium phosphate, and 4 mmol/L ammonium molybdate) in eppendrof tubes. After closing, were placed in water bath for 90 min at 95°C and the absorbance of samples was measured at 695 nm. The control sample, 1 mL of solvent was used instead of extract.

### Preparation soybean oil contains *Cressa cretica* ethanolic extract

In order to evaluate antioxidant activity of the extract in oil, deodorized and bleached soybean oil with no additives was used. *Cressa cretica* ethanolic extract in six level (200, 400, 600, 800, and 1000 ppm) and synthetic antioxidant (BHT, 200 ppm) were added to oil, then mixed by magnetic heater (Fater Electronics) in 30 min. soybean oil with no additives was used as control sample. The samples were transferred into a series of glass and incubated for 16 days at 63°C.

### Peroxide value

The peroxide value (PV) was determined according to the official methods of AOAC (1990). Briefly, the oil sample (3 g) was dissolved in 30 mL glacial acetic acid‐chloroform (3:2 v/v). Then, saturated KI solution (1 mL) was added. The mixture was kept in the dark for 1 min, after adding of distilled water (30 mL), the mixture was titrated against sodium thiosulfate (0.01 N). Titration was continued almost until disappearance of yellow color. Then, 5.0 mL of starch indicator was added and continued the titration until the blue color disappears. The PV value (mEq of oxygen/kg) was calculated using the following equation:

PV value = (V_2_‐V_1_) × N × 1000/M.

where V_1_ and V_2_ are the titrated volume of sample and control in ml (respectively), N is the normality of sodium thiosulfate solution, and M is the weight of oil sample (gr).

### Thiobarbituric acid value

Thiobarbituric (TBA) acid assay to measure secondary oxidation products of malonaldehyde was performed according to AOAC (1990) procedure. Oil sample (200 mg) was dissolved in a small volume of 1‐butanol and made up to volume with the same solvent (25 mL), then 5.0 mL of this solution was mixed with 10 mL of TBA reagent (0.2%), incubated for 2 h at 95°C water bath and cooled under running tap‐water for about 10 min until room temperature. The absorbance was measured at 532 nm against a blank (reaction with all the reagents except the oil) and TBA value was calculated by the following equation:

TBA value = 50 × (A‐B)/m.

where A is the absorbance of the test solution, B is the absorbance of the reagent blank, and m is the weight of oil sample (mg).

### Statistical analysis

Each measurement was performed at least in triplicate. The data obtained were analyzed by running one‐way analysis of variance (ANOVA) using SPSS software version 18.0. A one‐way ANOVA was used to evaluate difference in the mean value of samples and control. All mean separations were performed by Duncan multiple range test using the significance level of 95% (*P *< 0.05).

## Results and Discussion

### The total phenolic contents

The choice of solvent to extraction of phenolic compounds depends on the target compound solubility, the interaction between the solvent and plant material, and factors such as constant and dielectric loss (Wang and Weller 2006). Ethanol and/or methanol solvents have a lower dielectric constant than water, but when used with water as a solvent, a better result is achieved. Ethanol solvent due to good ability to solve phenolic compounds is better than water solvent (Zhang et al. 2010). Time also had significant effect on extraction of phenolic compounds because during storage, the solvent gives a chance to penetrate into the plant tissue, as well as phenolic compounds are also ample opportunities to get away from the matrix and enter to the solvent (Shahidi 1997; Pinelo et al. 2005).

In this study, the total phenol compounds were extracted by the water and ethanol 70% solvents from 3 to 24 h to select the optimum extraction condition. Comparison of different treatments results were shown that the effects of the solvent type, time and interaction of these parameter were significant (*P *< 0.05) (Table 1). The extraction of phenolic compounds by the two solvents within 3–18 h were enhanced, while after that time, total phenolic compounds mount were reduced. The highest total phenolic compounds mount were extracted by the ethanol 70% solvent within 18 h (68.512 ± 0.36^ ^mg gallic acid/g dry extract), while the same time, water solvent had the lowest performance in extraction of phenolic compounds by immersion method (Table 1). Pryianka et al. (2015) were reported high phenolic content, that is, 99.09 ± 0.10 *μ*g/mg of petroleum ether extract of *Cressa cretica*. Also, the TPC of ethylacetate and methanolic fractions of *Cressa cretica* were obtained, respectively, 7.081 ± 1.033 and 12.833 ± 0.24 mg gallic acid equivalents (GAE)/g dry extract by Sunita et al. (2011). Comparing these results were exhibited that the solvent type, extraction time and location of plant growth were effective on the total phenolic content of *Cressa cretica*. Franco et al. (2008) were shown that the highest phenolic compounds extracted from *Rosa rubiginosa* and *Gevuina* with various solvents was related to the ethanol solvent compared with water and methanol solvents (Franco et al. 2008). In addition, high levels of phenolic compounds from alcoholic extract than to aqueous extract of *Hieracium pilosella* L. plant were reported in research of Stanojević et al. (2009). In another study, with increase in extraction time over 20 h, Pinelo et al. (2005) were faced with a reduction in phenolic compounds content, which were caused by thermal decomposition or polymerization. These results were in agreement with our findings.

**Table 1:** The effects of the solvent type and time on the phenolic compounds extraction (mg gallic acid/g dry extract) of *Cressa cretica*

Time (hours)	Solvent	
	Ethanol 70%	Water
3	62.798 ± 0.12^a^	42.235 ± 0.23^a^
6	62.108 ± 0.21^b^	42.149 ± 0.59^a^
9	64.611 ± 0.38^c^	43.210 ± 0.24^b^
12	67.371 ± 0.27^d^	43.517 ± 0.27^c^
18	68.512 ± 0.36^e^	45.412 ± 0.21^d^
21	63.218 ± 0.31^f^	45.176 ± 0.36^d^
24	63.171 ± 0.5^f^	42.651 ± 0.17^e^

Values in the same column followed by different letters are significantly different (*P *<* *0.05).

### DPPH radical scavenging activity

**Figure 1:**
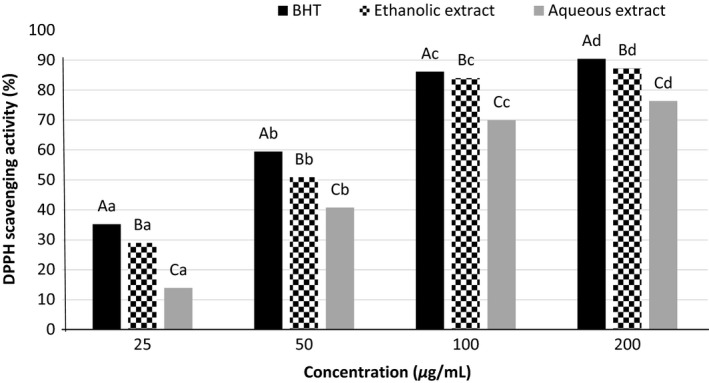
1,1‐diphenyl‐2‐picryl hydrazyl radical scavenging activities of the ethanolic and aqueous extracts of *Cressa cretica* and BHT. Differences between small letters indicate significant differences in different concentrations of a certain sample (*P* ≤ 0.05). Differences between capital letters indicate significant differences between the samples in a certain concentration (*P* ≤ 0.05).

DPPH scavenging activity assay is widely used to evaluate the ability of compounds to scavenge‐free radicals or donate hydrogen/electron, and determine the antioxidant activity in foods (Bidchol et al. 2011). DPPH assay is measured substantially water‐soluble phenolic antioxidant activity, so obtained similar results of the two extracts antioxidant activity by the DPPH method indicate that they have the same hydrophilic molecules (Chun et al. 2005). Different extracts show different antiradical activity due to the types and contents of their polyphenolic compounds and other phytochemical constituents. Also, hydroxyl group position, the presence of other functional groups such as double bonds and the composition of the hydroxyl groups and ketone groups plays an important role in antioxidant activity (Memon et al. 2007). Figure 1 is shown the DPPH^•^ scavenging activity of *Cressa cretica* ethanolic and aqueous extracts compared to synthetic antioxidant namely BHT. The type and concentration of the extracts and synthetic antioxidant had a significant effect on DPPH^•^ inhibition percentage (*P *< 0.05). Free radical scavenging activity was enhanced by increasing concentration of the extracts and BHT, due to the increase in phenolic compounds contents, so that the highest and lowest amounts of DPPH^•^ free radical scavenging activity were related to 200 and 25 ppm concentrations in all them, respectively. At all concentrations, BHT were shown higher inhibition than the water and ethanol extracts. Also, the ethanolic extract of *Cressa cretica* had higher inhibitory activity than to the water extract (Fig. 1). The results were in agreement with previous studies on *Cressa cretica* which reported a significant correlation between concentrations of plant extract and percentage inhibition of free radicals. For example, the petroleum ether extract of *Cressa cretica* with increasing concentrations (25–250 ppm) was revealed more DPPH^•^ scavenging activity (36.26–65.93%), and also it was shown weaker scavenging activity than to ascorbic acid. Of course, those findings of the petroleum ether extract from *Cressa cretica* was exhibited the lower percentage inhibition of DPPH^•^ compared with our results of the ethanolic extract (ranged of 28.98–87.29%) (Fig. 1). The same results were obtained in the ethylacetate and methanolic extracts of *Cressa cretica* (Verma et al. 2013). Pereira et al. (2009) were found similar results in evaluation of DPPH scavenging activity of *Melissa officinalis*. They found that the ethanol extract had the highest DPPH scavenging than the aqueous and methanol extracts (Pereira et al. 2009). These results were in agreement of our findings.

**Figure 2:**
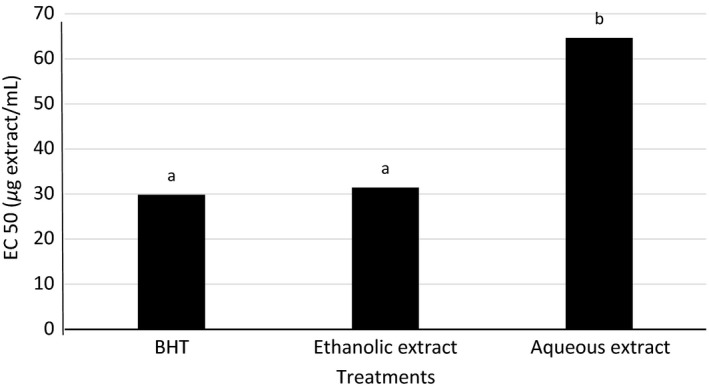
EC_50_ of the different extracts of *Cressa cretica* and BHT using 1,1‐diphenyl‐2‐picryl hydrazyl radical scavenging assay. Values followed by different letters are significantly different (*P*<0.05).

EC_50_ values of the aqueous and ethanolic extracts, and synthetic antioxidant were determined in order to compare them more accurately in terms of ability to inhibit free radicals DPPH. EC_50_ values denote the concentration of sample which is required to scavenge 50% of initial DPPH free radicals. So, lower EC_50_ value indicates higher antioxidant activity of samples. The difference in EC_50_ values is due to the difference amounts of phenolic compounds of samples. Researchers reported that samples with higher amounts of polyphenols had lower EC_50_ (Chun et al. 2005). As indicated in Figure 2, the aqueous extract was shown higher EC_50_ (64.63 *μ*g/mL), whereas there was no significant difference between EC_50_ of the ethanolic extract (31.44 *μ*g/mL) and BHT (29.87 *μ*g/mL). As a result, the ethanolic extract has antiradical activity more than the aqueous extract. EC_50_ value of petroleum ether extract from *C. cretica* was reported149.38 *μ*g/mL by Pryianka et al. (2015). Whereas, Sunita et al. (2011) were obtained EC_50_ values of the ethyl acetate and methanolic extracts of *C. cretica*, respectively, 48.72 *μ*g/mL and 92.56 *μ*g/mL. This is mean that the ethanolic extract had DPPH‐free radical scavenging more than to *C. cretica* methanolic, petroleum ether, ethyl acetate, and aqueous extracts. These different of EC_50_ values may be due to solvent type, extraction time, and grow place of the plant.

### Ferric reducing antioxidant power

This assay was based on the reduction in Mo (VI) to Mo (V) by the sample and the subsequent formation of a green phosphate/Mo (V) complex at acidic pH. Reducing properties are generally related to the ability of reductants to donate a hydrogen atom or electron and thereby break a radical chain (Ramarathnam et al. 1997). Thus, samples with higher reducing power are more able to donate electron and hydrogen atom.

**Figure 3:**
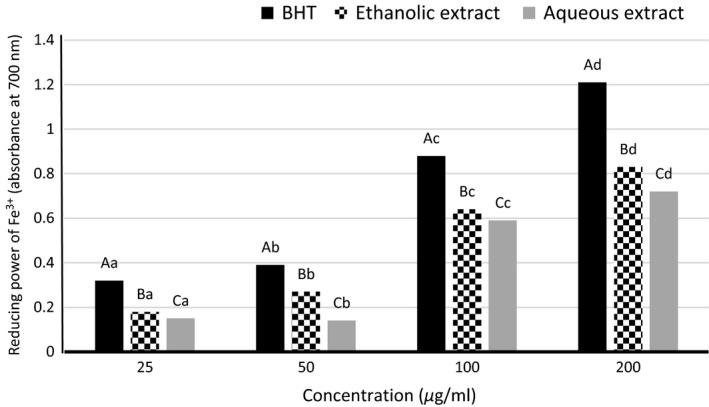
Reducing power of Fe^3+^ in different extracts of *Cressa cretica* and BHT.

The Fe^+3^ reducing power of different samples are shown in Fig. 3. Generally, the results were proved that the type and concentration of the extracts had a significant effect on reducing power (*P* < 0.05). Regarding the treatments, the reducing activity trend was as follow:

BHT > ethanolic extract> aqueous extract.

The reason may be due to nonspecific activity of ethanol solvent in the extraction of phenolic compounds. For this reason, along with phenolic compounds, other components also inter to the plant extracts. Some of these compounds can act as an electron and/or hydrogen atom donor agents, and using higher percentage of converting Fe^+3^ to Fe^+2^ increases the absorption rate of the solutions (Ghaderi ghahfarokhi et al. 2012). Also, increasing concentration of 25–200 ppm in both extracts and BHT was led to increasing in reducing power, which related to increasing total phenolic compounds content (Fig. 3). Similar result was also observed by Gramza et al. (2006) who examined the antioxidative activity of the water and ethanol extracts of green and black tea leaves against the oxidation of heated sunflower oil and lard, and found higher reducing activity of the ethanol extract than to the aqueous extract (Daraei Garmakhany et al. 2008). Pereira et al. (2007) were evaluated antioxidant activity of six varieties of walnut leaves aqueous extract and reported that the reducing power of the extracts were dependent on the concentration of which was in accordance with our results. Sunita et al. (2011) were investigated reduction potential of *C. cretica* ethyl acetate and methanolic extracts and compared to ascorbic acid. They were obtained reducing power in the order: ascorbic acid > Fr‐Et>Fr‐Me. Also, reducing potential was enhanced with increasing concentration. These findings were consistent with our results.

In order to compare of reducing ability of samples can be used a factor as EC_50_ value. In this method, the EC_50_ value represents the concentration of sample which is required to reduce 50% of Fe^+3^ ions at 700 nm. A lower EC_50_ indicates higher reducing power and antioxidant activity. Table 2 is shown the EC_50_ values of the ethanolic and aqueous extracts of *C. cretica* and BHT. EC_50_ value of the aqueous extract (200.94 *μ*g/mL) was significantly higher than BHT (41.79 *μ*g/mL) and the ethanolic extract (110.840 *μ*g/mL). As a result, the ethanolic extract had higher reducing potential than to the water extract (Table 2).

**Table 2:** The EC_50_ (*μ*g extract/ml) values in reducing power assay and total antioxidant capacity

Treatments	EC_50_	
	Reducing power assay	Total antioxidant capacity
Ethanol extract 70%	110.840 ± 0.377^a^	90.496 ± 0.83^a^
Aqueous extract	200.94 ± 0.045^c^	142.726 ± 0.57^b^
BHT	41.793 ± 0.045^b^	87.743 ± 0.68^a^

Values in the same column followed by different letters are significantly different (*P *< 0.05).

### Total antioxidant capacity

**Figure 4:**
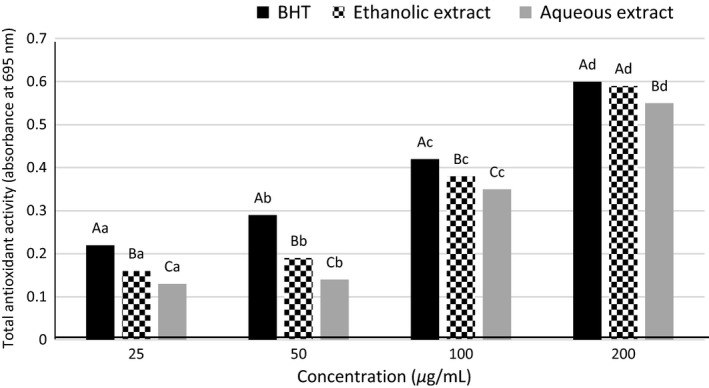
Total antioxidant activities of the *Cressa cretica* extracts and BHT.

Extracts with higher electron donating activity can terminate the radical chain and turn free radicals into more stable products (Sahreen et al. 2010). The extracts in this study were shown to have considerable amounts of phenolic compounds that can donate electrons. However, each of the extracts was behaved differently from the others, no doubt due to differences in solvent type and concentrations. Generally, the result of total antioxidant capacity was in the order: BHT>ethanolic extract>aqueous extract (Fig. 4). Comparison of the total antioxidant capacity of the ethanolic extract and synthetic antioxidant was found a significant difference in all levels except the concentration of 200 ppm (*P* < 0.05). Also, the total antioxidant capacity of the ethanolic and aqueous extracts, and BHT were increased gradually with increasing concentration of 25–200 ppm (Fig. 4).

In this method, the EC_50_ value is defined as the amount of extract necessary to decrease the initial 50% absorbance at 695 nm. Lower EC_50_ value signifies higher total antioxidant activity. According to the result, the order of EC_50_ values in this assay was BHT<ethanolic extract<aqueous extract (Table 2). These finding were proved order of total antioxidant capacity mentioned above.

In study of Arabshahi‐Delouee and Urooj 2007; total antioxidant capacity of the methanol, acetone, water extracts of mulberry leaves and BHT were obtained 1.393, 1.386, 0.66, and 3.921 tocopherol equivalent/g of extract, respectively, that indicated a direct correlation between the amount of phenolic compounds and total antioxidant capacity. Sahreen et al. (2010) and Sun et al. (2011) were reported relationship between the concentration and antioxidant capacity that were consistent with the results obtained in this study.

### Evaluation of antioxidant activity of the ethanolic extract of *Cressa cretica* in delaying the oxidation of soybean oil

Because the ethanolic extract of *Cressa cretica* was revealed antioxidant activities better than the aqueous extract, it was selected to evaluate in soybean oil. Soybean oil is used in this research due to the high percentage of unsaturated fatty acids (90%) and high oxidation capability. Since the oxidation of lipids is a complex process, using several methods of evaluating the oxidation process of vegetable oils is necessary, especially when consider the effectiveness of antioxidants such as herbal extracts. In this study, performance of *Cressa cretica* ethanolic extract to prevent oxidation of soybean oil was investigated by incubating at 63°C and measuring of the peroxide and TBA values during 16 days.

### Peroxide value

**Figure 5:**
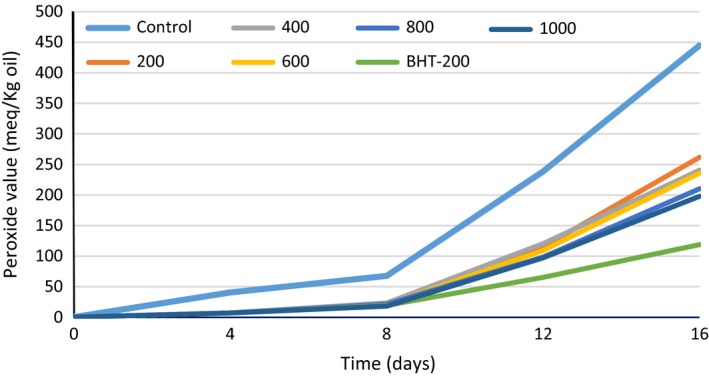
Peroxide value changes of different treatments of *Cressa cretica* ethanolic extract and BHT during 16 days at 63°C.

Peroxide value represents primary reaction products of lipid oxidation, which can be measured by their ability to liberate iodine from potassium iodide (Nor et al. 2008). In fact, the increase in the amount of PV can be attributed to the higher formation of hydroperoxides. In this work, PV was measured in samples including the control (without any antioxidant), 200 ppm of BHT (BHT‐200) and concentrations of 200–1000 ppm of *Cressa cretica* ethanolic extract. According to Figure 5, the PVs of all treatments have been rising over time, whereas the control sample (without any antioxidant) had the greatest increase in terms of PV, and also had significant difference with the other samples (*P *< 0.05). Because, the control sample had not any antiradical compounds for stopping of oxidation process. On the other hand, samples contain higher concentrations of the extract had the more inhibitory effect against oxidation due to present more phenolic compounds. Thus, oxidation was delayed in all samples except in the control treatment. Until eighth day, the lowest PV was attributed to sample contain 1000 ppm of the ethanolic extract and after that the lowest PV was found in BHT‐200 treatment (Fig. 5). These results were in agreement with Azeez et al. (2013) and Heo et al. (2007).

### Thiobarbithoric acid

The Thiobarbithoric acid (TBA) test determines the amount of malondialdehyde (MDA), a major secondary by‐product of lipid oxidation in food samples. In general, the greater unsaturated degree of oils leads to more prone for oxidation. When the PV reach a certain amount, volatile aldehydes and ketones will generate that effect on unpleasant odor, taste, and increase TBA value (Taghvaei et al. 2014). So at the end of storage, malondialdehyde was produced form peroxide decomposition which represents the second stage of oxidation. Then, in the early days of storage, TBA was low, and during storage with increasing amount of primary products and their decomposition, TBA value increases (Farag et al. 2007; Mohamadi et al. 2014; Mohammadi et al. 2016).

**Figure 6:**
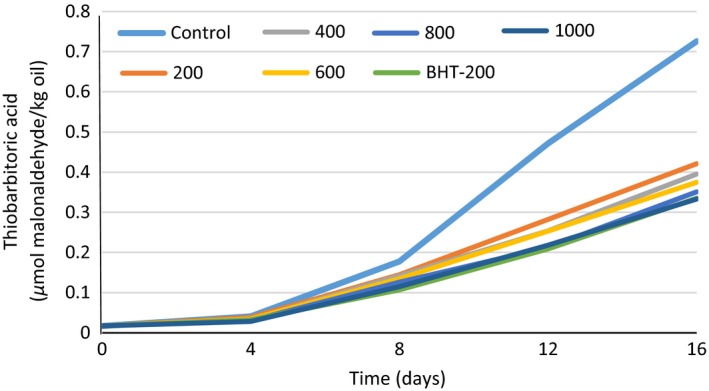
Thiobarbituric acid value changes in different treatments of *Cressa cretica* ethanolic extract and BHT during 16 days at 63°C.

In this work, TBA value was measured in samples including the control (without any antioxidant), BHT‐200 and concentrations of 200–1000 ppm of *Cressa cretica* ethanolic extract (Fig. 6). Based on our results in Fig. 6, at the start of the study, TBA values of different samples influenced by storage time were minimum amount and had fewer differences. However, that were increased significantly (*P* < 0.05) in all the days of storage. The control sample had the highest level of TBA values, whereas the lowest level until the fourth day was related to 1000 ppm of the ethanolic extract, between 4 and 12 days of storage was related to BHT‐200 sample and on 16th day was again related to 1000 ppm of the ethanolic extract (Fig. 6).

Farag et al. (2007) were obtained similar results by evaluating of olive leave extract (800 ppm) on oxidative stability of sunflower oil. Mohamadi et al. (2014) were demonstrated that Melissa officinalis methanolic extracts consist of phenolic compounds and rosmarinic acid had high antioxidant activity, and it could to cause soybean oil stability. The results also were in agreement with Buta et al. (2013) who studied the effect of lemon balm (Melissa officinalis) extract (200, 600, and 1000 ppm) compared with BHT regarding the retarding lipid oxidation of sunflower oil. They found that the lemon balm extract showed a significantly inhibitory effect on lipid oxidation during heat treatment. Lemon balm extract in dose of 200 ppm inhibited the lipid oxidation in a similar manner to BHT and in doses of 600 and 1000 ppm resulted in significant decrease in investigated indices (Buta et al. 2013). These findings were in compromise with our results.

## Conclusions

The aim of this study was extraction of phenolic compounds from *Cressa cretica* by immersion extraction method and different solvents (water and ethanol 70%) in different times. Total phenolic contents of the extracts were determined according to the Folin–Ciocalteu method, and the antioxidant activities of them were investigated by DPPH radical scavenging, ferric reduction activity potential (FRAP) and total antioxidant capacity assays and compared to synthetic antioxidant namely BHT. The results were proved that solvent type and time were effective on phenolic extraction efficiency. The ethanol solvent had phenolic extraction efficiency more than the water solvent. Increasing in time until 18 h was enhanced extraction of phenolic compounds, and afterward decreased. Therefore, the most total phenol content was outcome of the ethanolic extract after 18 h extraction (68.512 ± 0.36^ ^mg gallic acid/g dry extract). The results of DPPH radical scavenging, Fe^+3^ reducing power, and total antioxidant capacity assays as well as their EC_50_ values were indicated higher antioxidant potential in the order: BHT>ethanolic extract>aqueous extract. Also, the antioxidant activities were dependent to increasing concentration of the extracts and BHT, due to enhancement of phenolic content. Finally, the ethanolic extract with best efficiency was added to soybean oil (200, 400, 600, 800, and 1000 ppm) and compared by adding BHT synthetic antioxidant (200 ppm) and the control sample (without antioxidant). The results of PV and thiobarbituric acid tests were revealed that concentration of 1000 ppm of the ethanolic extract had the most effective to inhibit oxidation, so could be successfully replaced by BHT.

## Conflict of Interest

None declared.
